# SDN Architecture for 6LoWPAN Wireless Sensor Networks

**DOI:** 10.3390/s18113738

**Published:** 2018-11-02

**Authors:** Marcio L. F. Miguel, Edgard Jamhour, Marcelo E. Pellenz, Manoel C. Penna

**Affiliations:** PPGIa, Pontifical Catholic University of Parana, Curitiba 80215-901, Brazil; marcio.miguel@ppgia.pucpr.br (M.L.F.M.); jamhour@ppgia.pucpr.br (E.J.); marcelo@ppgia.pucpr.br (M.E.P.)

**Keywords:** software-defined wireless sensor network, 6LoWPAN, smart grid, advanced metering infrastructure, low power and lossy networks, neighborhood area network

## Abstract

Wireless sensor networks (WSN) are being increasingly used for data acquisition and control of remote devices. However, they present some constraints in critical and large-scale scenarios. The main limitations come from the nature of their components, such as lossy links, and devices with power supply limitations, poor processing power and limited memory. The main feature of software-defined networks (SDN) is the separation between the control plane and the data plane, making available a logically unified view of the topology in the controllers. In this way, it is possible to build network applications that take into account this unified view, which makes the SDN an alternative approach to solve the mentioned limitations. This paper presents the SD6WSN (software-defined 6LoWPAN wireless sensor network) architecture, developed to control the behavior of the data traffic in 6LoWPAN according to the SDN approach. It takes into account the specific characteristics of WSN devices, such as low data transfer rate, high latency, packet loss and low processing power, and takes advantage of the flexibility provided by flow-based forwarding, allowing the development of specific networking applications based on a unified view. We provide a detailed description of how we have implemented SD6WSN in the Contiki operating system. The new architecture is assessed in two experiments. The first considers a typical advanced metering infrastructure (AMI) network and measures the overhead of SD6WSN control messages in configurations involving different path lengths. The results indicate that the overhead introduced is not excessive, given the advantages that the SDN approach can bring. The second considers a grid-topology to evaluate the average latency of the peer-to-peer communication. It was observed that the average latency in the SD6WSN is considerably lower than that obtained with standard 6LoWPAN, showing the potential of the proposed approach.

## 1. Introduction

Due to simplicity and low energy consumption, wireless sensor networks (WSN) are are being increasingly used for data acquisition and control of remote devices. In general, WSN can be considered as a low power and lossy network (LLN), presenting some constraints for use in critical and large-scale scenarios. The main limitations come from the nature of its components, such as lossy links, and devices with power supply limitations, poor processing power and limited memory. Other important limitations are the difficulty of building WSN management systems and the lack of flexibility regarding policy changes [1]. The first prevents deployment in scenarios where the operation is subject to requirements that are more strict concerning reliability and performance, while the second stems from the rigidity of WSN with respect to policy changes, pointing to the difficulty to adapt it to factors external to communication and networking, such as business rules and accessibility.

A very important scenario with the aforementioned characteristics is the advanced metering infrastructure (AMI) of the smart grid. A relevant part of the AMI network is to handle communication in the neighborhood area, which usually involves many metering devices deployed in a large and complex geographical area. AMI requires that readings of thousands of metering devices be routed to a central collector and the execution of remote commands, such as cut-off and reconnection of consumers. Considering these requirements, the use of wireless communication is almost unanimous for the collection of information and operation of AMI devices.

The possibility of direct and bidirectional access to devices using IP technology considerably reduces the mentioned difficulties, but some issues emerge with respect to the complexity of interconnection. A basic premise of IP is end-to-end communication, which allows devices to be accessed without the need for gateways to adapt protocols. The 6LoWPAN standard (IPv6 over low power wireless personal area networks) has been developed to avoid adaptation [2], making it possible to reach WSN devices with IPv6 addresses. Routing decisions in 6LoWPAN are made by distributed protocols, especially the routing protocol for low power networks and losses (RPL) [3]. Each device routes the data using the information computed by RPL. Although 6LoWPAN (with RPL) has several positive aspects, some constraints have been pointed out: the computational complexity of the protocol executed on devices with low processing power and the lack of a unified vision of the network, which can contribute to high costs for building management systems [4].

A software-defined network (SDN) is an architecture that aims to make networks agile and flexible. SDN’s goal is to improve network control by enabling network operators to respond quickly to changes in business requirements. Moreover, because it allows networking applications to reason about the network from a unified view of the topology, it can be considered as an alternative to overcome the above limitations. This main advantage adds to others, such as providing more flexibility in the introduction of new services, allowing for vendor independence and enabling faster innovation [5]. SDN uses a central mechanism to make routing decisions instead of the decentralized protocols. Initially adopted in wired networks, the SDN approach has been expanded to wireless networks such as Wi-Fi mobile networks [6] and software-defined wireless sensor networks (SDWSN) [1].

This paper presents an architecture called SD6WSN (software-defined 6LoWPAN wireless sensor network), to control data plane forwarding in 6LoWPAN according to the SDN approach. SD6WSN defines a controller that communicates with data plane nodes through a control plane protocol (SD6WSNP), which uses IPv6 and RPL at layer 3, UDP at layer 4 and the constrained application protocol (CoAP) [7] at the application layer. The controller uses SD6WSNP messages to send rules dictated by network applications to the nodes, which in turn define the forwarding of data plane packets. The maintenance of the RPL communication stack at the control plane allows the coexistence of SD6WSN nodes with conventional 6LoWPAN devices, allowing gradual migration, without discontinuity of the operating services.

SD6WSN allows network applications to implement routing algorithms in addition to RPL, overcoming the lack of flexibility in 6LoWPAN. A unified view enables network applications to solve complex problems. Traffic can be balanced through the routing nodes, allowing the optimized use of power and reducing interference and contention regions in the transmission medium. Algorithms can also control the radio interface, changing the transmission power to prioritize specific data streams, building routes that meet special quality requirements. Network applications can also build virtual networks to isolate traffic among private nodes, including mechanisms that enable them to meet application-specific security requirements. In addition, due to the unified view, centralized control enhances the planning and management capacity, facilitating the construction of management systems.

We evaluate SD6WSN in two experiments. The first was chosen due to the prominence of 6LoWPAN in the construction of AMI networks and neighbor area networks (NAN) of companies in the electric power sector [8], supporting bidirectional communication for a more effective control of the electricity distribution network. Among others, this includes measurement, load control (of consumers) and distributed photovoltaic generation. The second experiment evaluates communication between peer devices, a scenario that becomes increasingly important in the so-called Internet of Things (IoT). Our experiments confirm that the proposed approach provides performance gains when compared to 6LoWPAN.

The remaining of this paper is organized as follows: Section 2 discusses the related work, including the limitations pointed out in the literature to the 6LoWPAN-RPL approach and the differences of our approach with respect to others that adopt SDN techniques for sensor networks. In Section 3, we describe the architecture, extending the presentation of the SD6WSN control plane presented in [9]. In Section 4, we provide a detailed description of how we have implemented SD6WSN in the Contiki operating system, including the software options to adapt a constrained device to act as an SD6WSN node, and the hardware options for real implementations. Section 5 presents the two experiments that compare the performance of SD6WSN with respect to the 6LoWPAN-RPL approach. Section 6 presents the findings and final remarks.

## 2. Related Work

The goal of SD6WSN is to bring the SDN approach to 6LoWPAN, supporting the implementation of complex networking applications not supported by RPL. The last is an IPv6 routing protocol that specifies how to construct a routing topology based on a destination oriented directed acyclic graph (DODAG), using an objective function that defines how a node in a DODAG selects its parents according to the routing metrics [3]. The objective function computes a rank value, which represents the distance from the node up to the root with respect to a given metric. RPL uses the trickle algorithm [10] to react to changes in topology that, at steady state, sends control messages at low speed, and speeding up to resolve inconsistencies [11,12,13].

DODAG is best suited for converge-cast traffic, and when direct communication between sensors occurs, WSN would be subject to congestion in the links near the root. The authors in [4] point out difficulties regarding the possible fragmentation of RPL control messages and issues regarding the hypothesis of bi-directionality assumed for the links since their quality is only evaluated in the routes towards the root. They also point out that RPL does not guarantee the absence of loops, and even if it can repair a loop when it is detected, this increases latency. The performance of RPL in AMI networks, with the emphasis on the reliability and stability, is evaluated in [14]. The conclusion is that the protocol often chooses sub-optimized paths due to inefficient routing decisions, which is the main factor in decreasing the packet delivery rate. In a subsequent paper [15], the same authors analyzed the role of the RPL protocol in a broader context of smart grid networks. Although the same restrictions were mentioned, the authors concluded that the application of RPL is viable.

RPL uses the expected transmission count (ETX) [16] as one of the metrics to build the DODAG; however, it does not specify how to implement it. Several authors implemented measurement of ETX in the MAC layer [17,18], but this approach has a limitation. The calculation of ETX in the MAC layer can only be done when messages are sent in unicast mode. Because RPL constructs DODAG using control messages sent in multicast, the topology is built using pre-defined default values, which leads to the use of poor quality links. This difficulty was pointed out by Dawans et al. [18], who studied the problem of neighborhood management in high-density RPL networks. Another known limitation of RPL is the size of the parent set of a node in the DODAG. If the size is small and the quality of the links is variable, the DODAG will be unstable. When the measurement engine identifies that the ETX has increased too much, the link is considered not usable, causing the use of an alternate parent. However, when there are no alternative parents, the node sends messages requesting a parent, which can cause the entire subtree to be rebuilt below it. A parent set with a size too large inhibits a child node from selecting the best parent, leading to poor routing. This is because the rank of a node must be greater than the rank of any member of its set of parents [19].

RPL has also been compared to other alternatives. Kathuria et al. [20] presented a comparative study between RPL and AODV (ad-hoc on-demand distance vector protocol) in intelligent energy metering networks. The simulations concluded that the AODV presents inferior performance in terms of latency and packet delivery rate (PDR) with respect to RPL in networks with a large number of nodes. Herberg and Clausen [12] compared RPL with the LOAD (6LoWPAN ad-Hoc on-demand distance vector routing protocol), in large LLN networks with a predominance of bidirectional traffic. The authors argued that RPL is optimized for multipoint-to-point networks, with rare point-to-multipoint and point-to-point traffic. However, in AMI networks, there is a need for power meters or load controllers to receive commands frequently, and these types of traffic cannot be overlooked. The results showed that RPL provides less latency and that LOAD introduces less control traffic into the network. Yi et al. [21] compared RPL with the LOADng (lightweight on-demand ad-hoc distance-vector routing protocol), which was developed by the 6LoWPAN working group. The authors pointed out a very large dependency of RPL on the root node of the network, which can become a critical point in the case of traffic bottlenecks. The results also show a high tendency of loops and packet fragmentation in RPL.

Karaagnac et al. [22] presented a centralized control proposal for scheduling timeslots and channels for 6TiSCH networks using CoAP messages for the installation of schedule tables using the CoAP management standard interface (CoMI) and with messages encoded by concise binary object representation (CBOR) [23] to optimize the control messages’ length, following the YANG data model. Similar to our research, the observed CoAP notifications are used to monitor changes in topology and link quality with neighboring nodes. The changes in the read parameters cause the nodes’s schedules’ recalculation, with subsequent sending of these new schedules to the nodes involved in the changes through the 6TiSCH CoMI interface. Their approach is similar to ours, but the SD6WSNP also uses CoAP messages for the installation of entries in the flow tables to program data plane forwarding, according to the SDN approach.

Other studies propose the implementation of SDWSN in open-source platforms, such as TinySDN [24,25] and CoAP-SDAN [26]. TinySDN [24] is an SDWSN tool for the TinyOS operating system, where the topology and link quality information collected from the CTPprotocol are informed to the central controller through the ActiveMessageC TinyOS component. From this information, layer 2 paths were computed and installed in the nodes’ flow tables using the same messaging component. In a subsequent paper [25], the authors of TinySDN evaluated the change of the CTP protocol to RPL. The research on CoAP-SDAN [26] proposed employing CoAP messages to acquire network quality information from the nodes and to install the centrally-computed routes in the nodes. The paper presented a packet delivery ratio comparison between the AODV, DSDVand their proposal, in a simulation environment. No details about the implementations on real constrained devices or protocol descriptions were presented. Unlike these preceding papers, ours provides a detailed specification of the architecture, which includes the control plane protocol, the flow table structure, the forwarding mechanisms in the data plane, the topology discovery and flow control. Moreover, a comprehensive description of the implementation of the SD6WSN in Contiki OS is provided, plus the description of our functional testbed based on real motes.

## 3. SD6WSN Architecture

**Figure 1:**
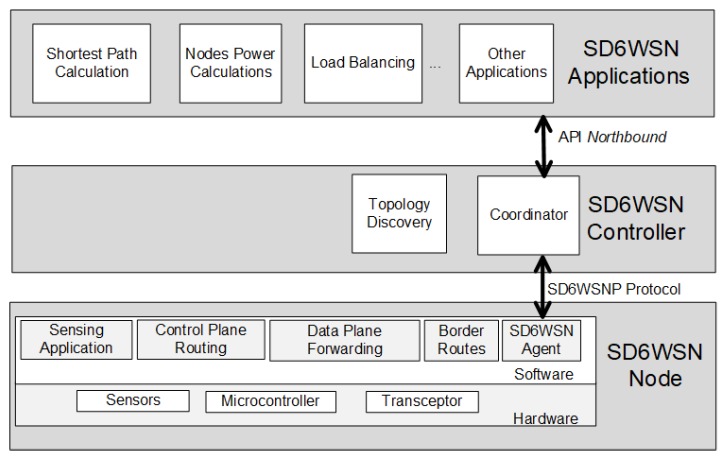
Software-defined 6LoWPAN wireless sensor network (SD6WSN) architecture.

SD6WSN is a typical SDN architecture, with a control layer composed of a set of controllers and a forwarding layer composed of a set of switching devices (in SD6WSN, we name switching devices as nodes). SD6WSN controllers include topology discovery and flow control functions, and the communication between controllers and nodes occurs through the southbound API. Networking applications connect to the controller to implement networking algorithms. Nodes are typical WSN devices extended with the following communication functions: border routing, data plane forwarding and control plane routing. The communication functions and sensing applications are coordinated by the SD6WSN agent, which interacts with the controller coordinator through the control plane protocol (SD6WSNP). Figure 1 depicts the SD6WSN architecture.

The IETF has established the working group WPAN to standardize a solution for IoT, which includes IEEE 802.15.4 at the MAC layer, 6LoWPAN and RPL at the network layer, UDP at the transport layer and CoAP at the application layer.

**Figure 2:**
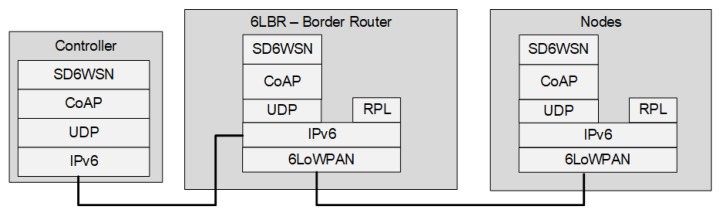
Communication between SD6WSN components.

SD6WSNP uses CoAP messages as the basis of communication, allowing the controller to perform the following operations: to manage entries in flow tables; to obtain local information from nodes including neighborhood, wireless link quality, geo-location and power transmission level; to modify node behavior, by setting power transmission level and transmission channel. Through SD6WSNP messages, the controller sends instructions to the agent (which has an internal CoAP server), which interprets the messages and performs actions, such as installing rules in the flow table. The agent also interacts with lower-level processes in the operating system (such as the TCP/IP stack) to drive packet forwarding according to the flow table. Some nodes can include the border router function, which is performed in 6LoWPAN-RPL by a special node called 6LBR (6LoWPAN Border Router). This node is responsible for topology management and for the conversion of the IPv6 to 6LoWPAN. Figure 2 presents the SD6WSN communication architecture.

The security issue is essential for applications running in SDN. For example, in OpenFlow, the switch and controller communicate through a transport layer security (TLS) connection, which is initiated by the switch on startup to the controller. In SD6WSN, communication at layer 4 uses the UDP protocol; thus, the datagram transport layer security (DTLS) is more appropriate to securitize communication. DTLS is a communication protocol that provides security for datagram-based applications, designed to prevent eavesdropping, tampering or message forgery.

The SD6WSN architecture extends the 6LoWPAN wireless sensor network, which can alone provide message delivery. The last uses 6LoWPAN at layer 3 and UDP at layer 4, and the routing protocol is RPL. SD6WSN uses this delivery stack as the end-to-end transport protocol for supporting control plane communication, that is to transport the messages of SD6WSNP. This is the way that flow-based SDN architectures transport control plane packets. For example, OpenFlow control messages can be encapsulated in TCP packets transiting a TCP-IP network, routed by some of the standard protocols (e.g., OSPF).

In flow-based SDN, the controller is in charge of routing decisions. It sends the necessary information to switches so that they can forward packets in the data plane accordingly. In other words, the controller drives packet forwarding by storing rules in flow tables present in switches. Packets that do not match any installed flow table entry are forwarded to the controller, which computes a path for the new flow and install an entry instructing how to forward the packets of the new flow in every switch along the computed path. If the controller decides to not route a packet, it installs a new entry instructing to drop it in the origin node. SD6WSN does the same: it uses the control plane protocol (SD6WSNP) to store in the nodes the rules that the drive communication in the data plane.

In SD6WSN, networking applications are responsible for solving possible connectivity problems. Thus, it is very important that the controller maintains the unified view of the network as close to reality as possible. To do this, it needs to have a continuous connection with the nodes, to be able to receive the notifications and to send corrections to the entries in the flow tables. SD6WSNP messages are transported by a reliable protocol stack (6LoWPAN-RPL), assuring that the information of every node is continuously sent to the controller, which in turn can continuously update the contents of the flow tables, according to the decisions made by networking applications.

Flow tables are composed of two fields: match, where the characteristics of the incoming packet header that identifies the corresponding flow are defined, and action, where the forwarding behavior for a matching package is defined. A reduced set of attributes (with respect to standard SDN flow table entries) is included in the match field: IPv6 source address, IPv6 destination address, TCP source port, TCP destination port, UDP source port and UDP destination port. Each entry has a unique identifier, ranging from one to 255. Because the attributes can contain wild-cards, the header of an incoming packet can match multiple entries in the flow table. When this occurs, the more specific entry, that is the one that matches more non wild-card attributes, has the higher priority. When there are multiple entries with equivalent matching, the tiebreaker is the identification number of the flow entry (flowid), the lowest having the highest priority. IP address aggregation techniques can use the destination IP address mask (dstmask)to decrease the number of entries in the tables. If this field is not specified, mask/128 is assumed. Table 1 describes possible attributes in SD6WSN flow table entries.

**Figure 3:**
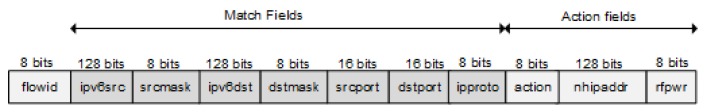
Flow table entry format.

The action field has three attributes: type, nhipaddr and rfpwr. The first defines the kind of action to be taken, the second the is the next-hop address in data plane forwarding and the third is the transmission power to be adopted for specific flows. If not present, the default transmission power is assumed. Table 2 describes the attributes of the action, field, and Figure 3 shows the format of flow-table entries.

Messages of SD6WSNP are constructed over CoAP confirmed messages. The CoAP protocol was initially designed as a client-server protocol, with the communication initiatives taken by the client. It was later extended with the observe option [27], activated by a flag in GET messages sent by the client, which instructs the server to register the request and send notifications back under certain conditions. The controller assumes the role of CoAP client, while the nodes assume the role of server The observe option is used whenever a relevant change state occurs or when a node receives a packet that does not match any existing flow. Three events are considered relevant changes: node failure, significant improvement of the quality of a link and deterioration of the quality of a link. The first is identified when a percentage of one node’s neighbors fail to report it on their neighborhood lists. The change in the quality of links is evaluated by the ETX metric. This kind of change should be carefully evaluated because it is common for transient changes in the quality of the transmission medium in wireless networks. Therefore, the ETX metric is calculated by an exponential moving average. In all three cases, the relevance of the change is defined by percentage thresholds that are configurable, all having default values. A node is considered inoperative when more than 50% (default parameterization) of its neighbors fail to report it in their neighborhood lists. In the default parameterization, the deterioration of link quality is considered relevant when a 100% increase in the ETX metric occurs in relation to the last notification sent, and the improvement is considered relevant when a 50% reduction of ETX is reported. The format adopted for response and notification messages is JSON (JavaScript Object Notation) [28]. This format is largely used and has the advantage of a simple structure, with the delimitation of the fields formed by unique characters, like keys and brackets.

SD6WSNP defines four messages: node-mod, info-get, flow-mod and packet-in. Messages are classified according to the process to which they are associated. Node-mod and info-get are used for topology discovery and maintenance, while flow-mod and packet-in are used for flow control. Node-mod is the first message sent by SD6WSNP, from the controller to the border router (6LBR), requesting a notification every time a new node is identified by RPL. Just after receiving a notification, the controller sends info-get to obtain the neighbors of the discovered node and the quality of respective wireless links. A networking application can also ask the controller to send an info-get, whenever it needs information from a node. After registering the node information in its database, the controller sends a packet-in message instructing the node to send back a notification when it receives data plane packets that do not match any entry in its flow table. Flow-mod is used to insert and remove entries in flow tables, allowing the networking applications to install flows according to their traffic needs. Table 3 summarizes SD6WSNP messages, and more details are provided in [9].

### 3.1. SD6WSN Controller

**Figure 4:**
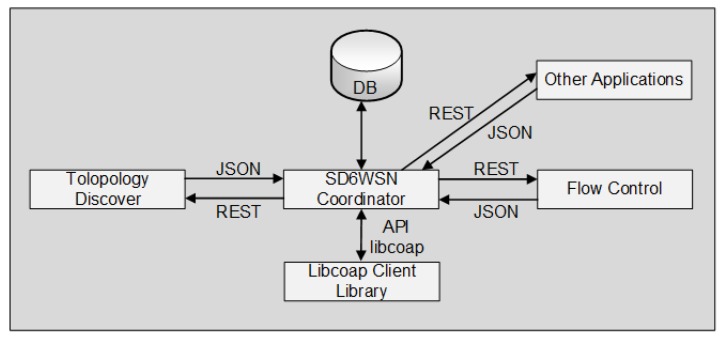
Diagram of the interaction of the controller modules.

The SD6WSN controller consists of three modules: coordinator; topology discovery and management; and flow control. The coordinator performs the integration with the networking applications. It uses the northbound interface to communicate with the applications the southbound interface for communication with the nodes. The topology discovery and management are triggered by flow-mod notifications and by info-get notifications received from nodes, informing significant changing in neighborhood wireless channels. The flow control module receives and process packet-in notifications. The coordinator sends and receives the SD6WSNP messages through the CoAP API of the Libcoap client library [29] and interacts with other modules and networking applications through REST and JSON messages, as shown in Figure 4. The state of the SD6WSN is stored in the networking database (DB).

#### 3.1.1. Topology Discovery and Management

To maintain the unified view of the network, the controller needs to identify the active nodes, but also the neighbors that each node sees, including the quality of the wireless links that connect them, which is indicated by ETX. The SD6WSNP protocol uses the information sent in flow-mod notifications to include in and exclude nodes from the SD6WSN topology. In the case of inclusion, the controller checks if the registered node is part of a preloaded table of authorized IPv6 addresses, sending to the node the info-get/nbr-etx message to obtain its neighbors and the respective quality of links (ETX). This message is sent with the observe option enabled, so that whenever a significant change in the quality of the wireless communication channels occurs, this can be notified to the controller. Upon receiving the information, the controller informs the changes to networking applications, which perform the recalculation of the existing flows and inform the controller of the necessary modifications. When a node is excluded, 6LBR notifies the controller, which warns the applications about the topology change, allowing them to decide if there is a need to recalculate the paths of existing flows. In some situations, such as a reboot of a node, it is possible for a CoAP observe record to be lost. To work around, the controller starts a timer when an info-get/nbr-etx message is sent to a node, which is restarted whenever a response arrives at the controller. If the timer exceeds the defined limit, a new info-get message is sent to the node in question. The controller can use the info-get message to request other information from nodes (e.g., battery status), which may be sent with the observe option if it is necessary to follow the variation of these parameters.

#### 3.1.2. Flow Control

**Figure 5:**
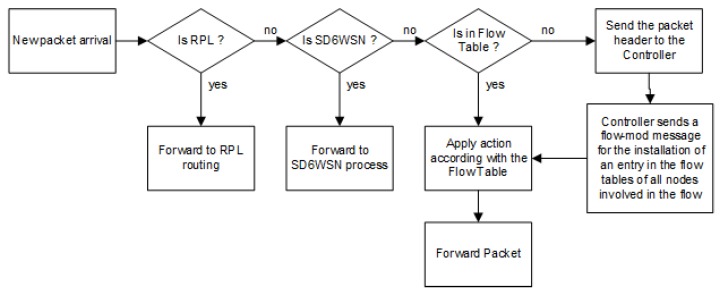
Flowchart for handling incoming packets in a node.

The flow-mod SD6WSNP message includes or removes entries in flow tables. If an entry already exists, it is overwritten. The installation of a new flow results from the decision of networking applications, as a consequence of topology change or topology change notification. When a packet arrives in a node, SD6WSN checks the IP header fields according to the matching rules in the flow table, looking for an existing flow. If a matching occurs, the node performs the associated action, that is it forwards the packet in the data plane or discards it (see Figure 5). Otherwise, the entering node sends a notification using the CoAP observe mechanism. Packet-in notification is formatted in a JSON message and contains the received packet header. When the controller receives the packet-in notification, it identifies networking applications, which calculate the corresponding route and the set of entries for the flow tables that will be inserted into/modified by nodes along to the path, to ensure the correct forwarding in the data plane.

### 3.2. SD6WSN Nodes

Each SD6WSN node has at least two components: agent and control plane routing. At least one node must include a third component named border router. A sensing component, which is application dependent, can be included in any node.

**Figure 6:**
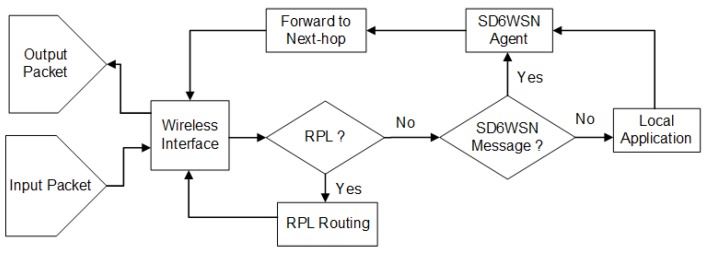
Packet forwarding procedure.

The agent is a CoAP server that communicates with the controller through SD6WSNP messages. It changes the local operating system packet transmission procedure (data plane forwarding) and interacts with the local operating system to read and modify specific mote information. The agent’s main functions are: (i) send information to the controller node; (ii) manage the entries in the flows table; (iii) inspect data plane packet headers; (iv) send or discard data plane packets according to matching entries in the flow table; (v) send packet-in notifications to the controller when there is no matching entry; and (v) interact with the operating system to change mote transmission parameters, such as transmission channel and RF power. SD6WSN modifies package forwarding at the operating system as follows. It inspects packets headers and checks if the incoming packet is an RPL message, and if so, it is routed through the control plane, that is it follows standard 6LoWPAN-RPL routing. Otherwise, if the packet contains an SD6WSNP message, it is handled by the agent. Otherwise, the packet is delivered to the local sensing application (see Figure 6).

The border router extends standard 6LBR with three functions: (i) notify the controller when a node is detected or removed in 6LoWPAN; (ii) perform the routing between the IPv6 and 6LoWPAN network; (iii) identify the packets belonging to SD6WSN flows and handle them properly. Nodes with the border routing function perform data plane forwarding as described in Figure 6.

## 4. Implementation of SD6WSN on the Contiki Platform

This section details how the components of SD6WSN are implemented in the Contiki operating system. Programming code, scripts for installing the development environment, and libraries are available on the SD6WSN repository site [30].

### 4.1. Software Architecture

**Figure 7:**
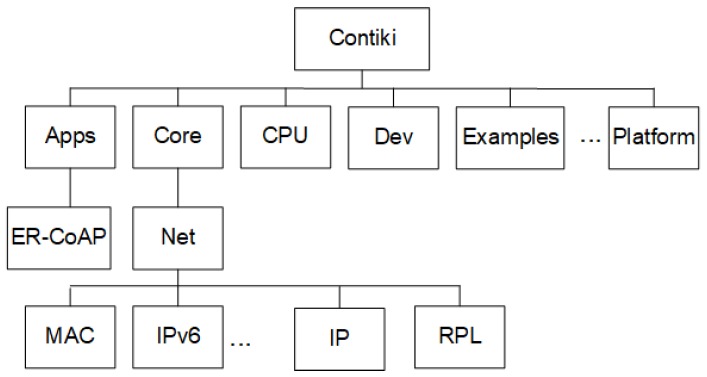
Directory tree of Contiki O.S.

Contiki [31] is an open source operating system specially developed for networked embedded systems, which provides an implementation of the IEEE802.15.4/6LoWPAN/RPL/CoAP stack for use in lossy networks with devices with little processing capacity and memory. Integration between the SD6WSN agent and Contiki takes place through the API provided by the operating system. The Contiki code is organized in a directory tree as shown in Figure 7. The “/core/net/” directory contains the link layer handling functions, including link quality metrics such as ETX calculation and RSSI measurements. The “/core/net/rpl” directory contains the code that implements RPL and “/core/net/ipv6” the code for IPv6 and 6LoWPAN. The directory ER-CoAP contains the default implementation of the ER-CoAP server. The “Platform” and “CPU” directories contain the specific functions for building firmware for the supported motes. Device drivers, such as radios and Ethernet adapters, are stored in the “Dev” directory.

**Figure 8:**
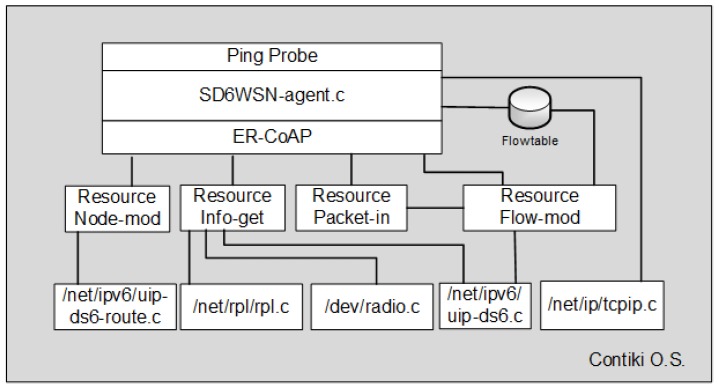
Integration of the SD6WSN agent with Contiki O.S.

The agent is implemented by the program “SD6WSN-Agent.c”, which integrates with the ER-CoAP server through function calls. It implements the functions that handle SD6WSNP messages (called resources). SD6WSNP messages are addressed to specific resources according to their URIs. In addition to implementing control plane messaging, the ER-CoAP server is responsible for handling the notifications. The time between two consecutive notifications is parameterizable and must be set so as to avoid a significant impact on the total traffic of the WSN. Figure 8 illustrates the integration between agent, ER-CoAP, resources and Contiki core libraries. One can also observe the ping probe component, whose function is to make Contiki calculate the ETX metric for every link between neighbor nodes. There is a mechanism for active link quality testing, called “RPL Probing”, which generates RPL control messages, encapsulated in ICMPv6 and transmitted in unicast mode, causing the update of ETX values in the corresponding links. However, this mechanism only sends messages to the preferred parents, leaving the other links without an update. The ping probe mechanism of the SD6WSN agent periodically sends ICMPv6 echo-request messages (the same used by ping) causing Contiki to return echo-reply messages and the consequent update of ETX in all links involved.

As mentioned before, the routing of SD6WSNP messages inside the 6LoWPAN-WSN is performed by RPL, but the routing of IPv6 packets to the controller and other network elements outside 6LoWPAN-WSN is performed by the standard TCP/IP stack. RPL routing on SD6WSN nodes is performed by the native Contiki RPL libraries, located in /core/net/rpl, and by the “border-router.c” application within 6LBR, which also uses the Contiki libraries to implement the 6LBR functions.

**Figure 9:**
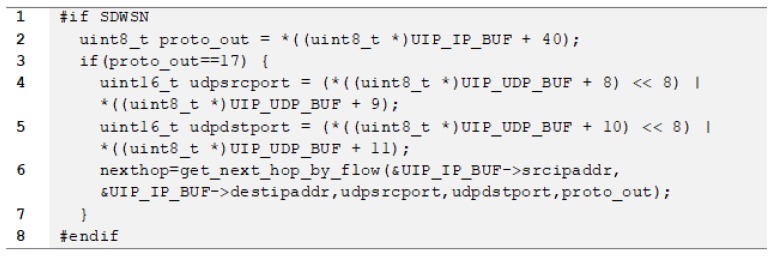
Next-hop address determination in Contiki’s “tcpip.c” program.

Packet forwarding logic is in the “core/net/ip” directory tree, especially in the “tcpip.c” program. We changed the packet processing flow by inserting the code snippet shown in Figure 9, which inspects the header fields of the incoming packet. If it is a UDP packet (Line 3), the source and destination ports are identified (Lines 4 and 5), and the get-next-hop-by-flow (Line 6) function (implemented in the agent) is called with the data of the header fields as arguments. The agent returns the IPv6 address of the next-hop.

The function get-next-hop-by-flow implements the forwarding logic. First, it looks up the entry that best matches the packet header in the flow-table. If no entry matches the packet-header, a packet-in notification is sent. Then, the function returns the IPv6 address depending on the action present in the matching flow table entry. It returns NULL when the packet is a control plane message or when it matches an entry with a Type 3 action (see Table 2), forcing RPL to compute the next-hop address. It returns a loopback address when the packet matches an entry in the flow table with a Type 2 action or when no entry matches the packet-header, causing the packet to be discarded. It returns the IPv6 address stored in the nhipaddr field of the flow table entry when the packet matches an entry with a Type 1 action (see Table 2). The next-hop variable (Line 6) is used by RPL forwarding code.

The SD6WSN is designed as shown in Figure 5. However, in the current implementation, the mote does not buffer packets that do not match any entry in the flow table. These packets, which raise packet-in notifications, are discarded due to the memory limitation of current devices that were used in the testbed and simulation. We understand that this limitation is temporary and that it soon will be overcome, allowing the implementation of packet-in buffer management. It is important to point out that the implementation without buffering does not cause errors, because the networking application will receive the packet-in notification, will add an entry to the flow table and the following packages belonging to the same flow will be forwarded in accordance with the controller’s decision.

### 4.2. Hardware Architecture

Devices with IEEE 802.15.4 transceivers, also called motes, are composed of an integrated circuit with a power amplifier and a micro-controller that includes the implementation of the MAC layer. Some manufacturers aggregate a radio to the micro-controller into a single integrated circuit, called SoC (system on a chip). The main features of motes are low cost and low power consumption, associated with low processing capacities, and small flash memory and RAM. Because of these limiting characteristics, they are classified as limited resource devices. In 2010, an IETF working group called Constrained RESTful Environments (CoRE) was created to propose a framework for applications in IP networks using devices with limited resources. In [32], motes are classified according to the size of RAM and flash (see Table 4). Class 0 devices do not have sufficient resources to run an IP stack, requiring proprietary protocol gateways to be connected to IPv6 networks. Class 1 devices are necessary to implement an IPv6 stack with security features such as DTLS. SD6WSN nodes can be implemented either in Class 2 motes or in Class 1 motes with at least 32 kbytes of RAM and 100 kbytes of flash memory.

Another relevant point for the hardware architecture is the configuration of the node that runs the border router function. In the following, we present the four main options for constructing a Contiki-based 6LBR, each with a different organization of the communication layers.

#### 4.2.1. 6LBR in a Linux System Connected to an IEEE 802.15.4 Contiki Mote

In the first option, a computer with the Linux operating system hosts the implementation of 6LBR in Contiki (“native-border-router.c”). Contiki communication functions (named μIPv6) include IPv6 and 6LoWPAN at the network layer; ICMPv6, TCP, and UDP at the transport layer; RPL routing; and CoAP at the application layer. An external mote is in charge of physical and MAC layers, usually an SoC that binds to the host through a serial connection. The mote consists of an SoC, which includes an IEEE 802.15.4 radio. Its micro-controller loads Contiki and “slip-radio.c”, the last being in charge of the serial connection to the host. The MAC and RDC (radio duty cycle) layer drivers are chosen according to the WSN specification. The serial connection, called SLIP (serial line IP), is described by the developers as being IEEE 802.15.4 over a serial line, because IEEE 802.15.4 frames are transmitted through the serial connection.

**Figure 10:**
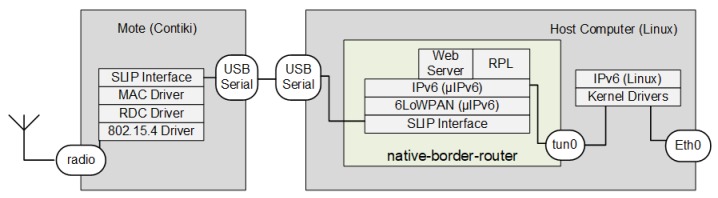
Linux host computer connected to a USB mote 6LBR option. SLIP, serial line IP.

The communication between the μIPv6 stack (in “native-border-router.c”) and IPv6 stack (in Linux) is performed through a tunnel interface (tun0). The command line defines the serial interface (SLIP) that “native-border-router.c” uses for communication with “slip-radio.c”. The Linux host must have an Ethernet interface for IPv6 communication outside 6LoWPAN. In Figure 10, one can observe two protocol stacks inside the Linux host, uIPv6 within “native-border-router.c” and the standard Linux IPv6 stack. The virtual interface (tun0) that provides communication between the two stacks is also depicted. A third communication stack can be seen in the 6LBR mote and its communication with “native-border-router.c” across the serial interface.

One of the advantages of this option is the possibility of using motes with less processing capacity and memory with the slip-radio function since only the physical, link and RDC (Radio Duty Cycle) layers are present. The layers that require more processing power are handled by the program running on the host computer, which usually has a processor far superior to those found in the motes.

#### 4.2.2. 6LBR in Full-Stack Contiki Mote Connected to a Linux System

**Figure 11:**
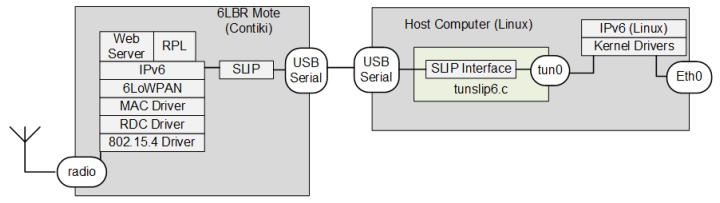
6LBR in a mote connected to a Linux host. tun0, tunnel interface.

In the second option, more 6LBR functions are displaced to the mote. There is a program named “border-router.c” program, where the layers 3 and 4 functions (6LoWPAN, IPv6 and RPL), as well as a simple HTTP server, are compiled together with layer 1 and layer 2 functions in “slip-radio.c”. Figure 11 shows this alternative that leaves in Linux only the program “tunslip6.c”, which is responsible for establishing a SLIP connection with the mote through a serial interface. Unlike the SLIP connection in the previous subsection, “tunslip.c” uses IPv6 packets to connect a Linux tunnel interface (tun0) directly to a SLIP process in the mote.

This option requires motes with better processors and with larger RAM and Flash, which deters the use of lower-cost motes. Even for more recent SoC, such as CC2538, there are still processing limitations. On the other hand, the host computer may be as simple as Wi-Fi routers running OpenWRT Linux distributions.

#### 4.2.3. 6LBR in a Full-Stack Contiki Mote with Ethernet Interface

**Figure 12:**
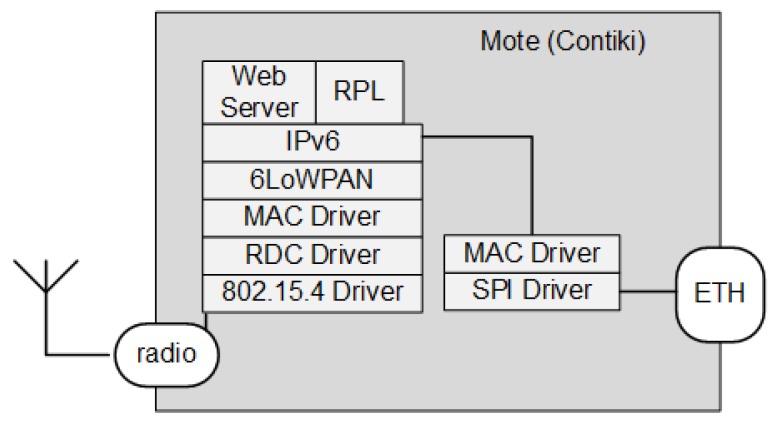
Complete 6LBR in a mote with Ethernet interface.

The third option is suitable when CPU and memory capacity cards are available, such as motes based on SoC CC2538, to which an Ethernet adapter with Serial Peripheral Interface (SPI) interface is coupled. This allows the 6LBR to be totally implemented in the Contiki mote. Because the SPI driver is directly connected to mote hardware, there is no need for a SLIP connection, as shown in Figure 12.

This option is becoming more common with the launch of SoCs with greater memory capacity, such as the SoC CC2538 integrated with an Ethernet module that uses the Microchip ENC28j60 controller. Despite the advantages of being an integrated solution, the disadvantage lies in the limited memory space for adding new components. Performance is also affected by the poor power of processing due to the inherent limitations of this class of low power devices such as the low clock frequency, for example 32 MHz for SoC CC2538.

#### 4.2.4. Native IEEE 802.15.4 and 6LoWPAN in Linux Kernels

Some projects, such as the “IEEE 802.15.4 Stack for Linux”, aim to implement the IEEE 802.15.4 and 6LoWPAN stacks for Linux, but are still in the “alpha” development stage. This option leaves all the processing of the MAC, RDC, 6LoWPAN, IPV6 and RPL layers to Linux. The external hardware only implements the physical layer, with the radio communicating with the computer by the SPI interface.

## 5. Evaluation and Results

In this section, we present the validation tests of SD6WSN. Functional assessment was performed in a real testbed and the performance evaluation in the COOJA/Contiki simulation environment. The use of COOJA allows collection of data, either by the log of the UART of the emulated motes or by the capture of the packets transported between them in the same format of real networks.

We performed two experiments to evaluate the 6LoWPAN network, with SDN (SD6WSN) and traditional (RPL) routing approaches. As in the other proposals of the SDN approach, ours uses the 6LoWPAN protocol stack as the message delivery protocol (in the control plane), just as the OpenFlow approach uses the TCP-IP protocol stack to deliver its control messages. The first experiment simulates a typical AMI network scenario in residential areas, with nodes distributed along a straight path on both sides of the street. Here, the multi-hop characteristic prevailed, with a high number of jumps between the 6LBR and the most distant node. The main objective of this experiment is to evaluate the overhead introduced by SD6WSNP, with respect to RPL. The goal is to compare the competing approaches in the communication scenario most favorable to RPL, that is communication traffic occurs only from nodes to 6LBR and vice versa. To evaluate the performance of our approach in this condition, we measured the overhead incurred by SD6WSNP messages and the end-to-end delay. We expected that the overhead would not be much higher, not excessively increasing network charge with the additional control messages, thus providing similar average latency. In the second experiment, we evaluate a different traffic model. We select a classic grid topology to evaluate network performance between peer-nodes. The goal is to compare the competing approaches in a communication scenario that tends to appear more frequently in solutions aimed at the Internet of Things, where this type of communication is expected. To compare the performance of the two approaches, we compare the average (one-way) communication latency between pairs of randomly-chosen nodes.

### 5.1. Evaluation Tools

#### 5.1.1. Validation Testbed

**Figure 13:**
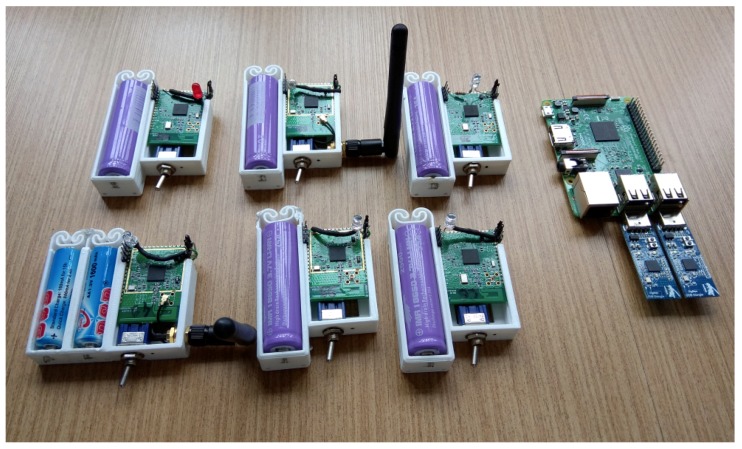
Test bed for the SD6WSN evaluation.

We used a simple test bed to validate SD6WSN in a real scenario based on motes of the CC25xx line (Texas Instruments). The environment features as a 6LBR a Raspberry Pi 3 model B minicomputer equipped with a USB mote that has the CC2531 chip and the Contiki firmware, compiled with the SD6WSN Border Router firmware. Figure 13 shows some components of the test bed, including a minicomputer with two motes, one being the USB Border Router and the second identical mote loaded with the CC2531EMK sniffer firmware, used to capture packets IEEE 802.15.4 by radio frequency, used for the real-time analysis of the packets transmitted by the nearby devices. The motes use the SoC Model CC2538 because they have adequate amounts of RAM (32 kbytes) and flash memory (512 kbytes) for the installation of the S.O.Contiki 3.0 and the SD6WSN agent.

#### 5.1.2. Simulation Environment

A sensor network using Contiki can be simulated using COOJA [33]. One of COOJA’s most important features is to allow simulation of a network, emulation of the operating system, as well as of machine code instructions [33]. This allows the configuration of networks with motes that emulate the operation of real hardware, including the firmware exclusively compiled for them. The Contiki-COOJA environment provides the image of a Linux virtual machine with the necessary software for compiling the firmware of several motes. The network topology can be defined and simulated with different options to capture the specificities of the wireless media. Data packets exchanged between motes can be captured and analyzed by the standard protocol analyzer (e.g., Wireshark) in the same way as in a real network.

We perform our experiments using “Wismote” motes, with 16 kbytes of RAM and 128 kbytes of flash memory, the MSP430 architecture and a 2.4-GHz CC2520 transceiver. We chose this mote type because its RAM is larger than others available in the COOJA simulator. The simulations were performed on a Linux Ubuntu 14.4 virtual machine on a computer with an Intel Core I7-6500U processor with 16 Gbytes of RAM. The environment contains the Contiki platform, including the compiler for the MSP430 and the COOJA simulator.

#### 5.1.3. Traffic Generation

A packet generation module included in the firmware simulates the sensing application. Packets are sent to a server installed in 6LBR, which receives and sends them back. The purpose is to generate traffic in the data plane, enabling the performance evaluation. The application sends UDP packets every 30 s, with random variation (uniform) of up to 5 s, to reduce collision probability. Packets have a 20-byte payload to prevent the frame size from exceeding 127 bytes, avoiding packet fragmentation. The simulation time is 20 min, allowing the application to send at least 30 messages per node.

### 5.2. AMI Scenario

**Figure 14:**
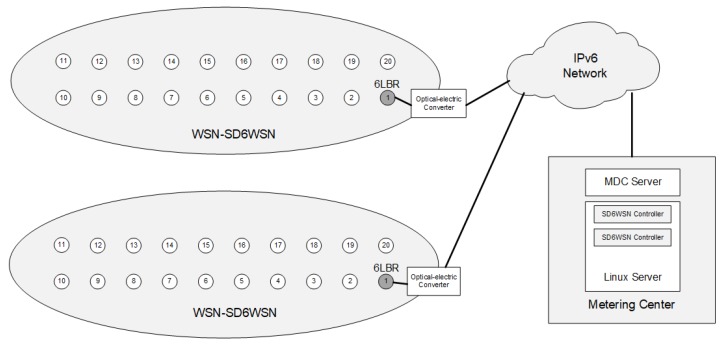
Advanced metering infrastructure (AMI) scenario. MDC, meter data collector.

In this scenario, we evaluate the communication between nodes and the 6LBR, the most common communication in 6LoWPAN-RPL networks. We compare the operation of an SD6WSN shortest path application against the RPL DODAG. The location of nodes is chosen to simulate a neighborhood area network (NAN) commonly found in AMI networks [34]. There are many real AMI scenarios that use 6LoWPAN for the communication between energy consumption meters and the meter data collector (MDC). The simulation scenario is a residential neighborhood formed by streets, with energy meters on both sides, using the grid-type topology shown in Figure 14. One can observe one 6LBR for each street connected to a metering center containing MDC servers and SD6WSN controllers. In this scenario, we evaluate the impact of SD6WSNP messages by comparing the number of such messages with the number of RPL control messages. We also evaluate the average round-trip latency observed in the paths computed by RPL with that observed in the paths computed by the SD6WSN application.

**Figure 15:**
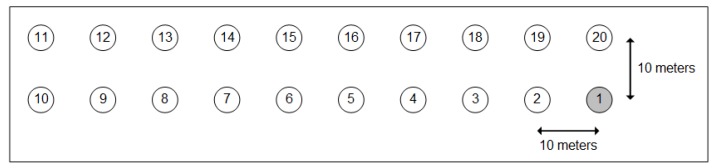
AMI simulations’ topology.

The grid is formed by two parallel lines with a distance of 10 m between nodes, where Node 1 represents the 6LBR (see Figure 15). This scenario allows variation of the number of hops in the paths by changing the transmission range of the nodes. In real scenarios, the range variation is due to several factors, among them the attenuation and reflections of the transmitted signal. The transmission range is varied using the Unit Disk Graph Medium (UDGM) model available on COOJA, setting transmission range to 25 m, 50 m, 100 m and 150 m and the interference range to twice the transmission range. The other wireless media settings in COOJA are transmission success rate to 75% and reception success rate to 100%.

**Figure 16:**
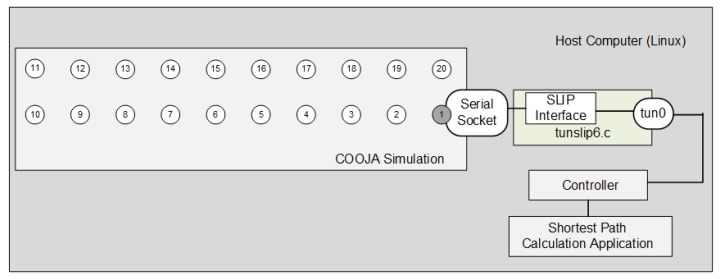
Integration between the COOJA simulation and the host computer.

As described in Section 4.2, the communication between the Contiki firmware emulated in COOJA and the hosting machine occurs through an SLIP connection provided by the program “tunslip6.c”. Figure 16 illustrates how COOJA integrates with the controller and the SD6WSN application. The firmware of 6LBR and motes also include the traffic generation application described in Section 5.1 and a server program that receives the traffic and returns it back. Traffic generation timings have been adjusted so that traffic starts 180 seconds after the initialization of the firmware, allowing RPL and SD6WSN processes to stabilize, with the entire topology discovered.

The paths between nodes and the 6LBR are computed for each transmission range, which directly influences the number of neighbors of each node. The RPL and the SD6WSN application use the ETX metric as the weight of the corresponding links, choosing the path with the lowest accumulated ETX. RPL uses a distributed protocol, while SD6WSN uses a centralized algorithm.

**Figure 17:**
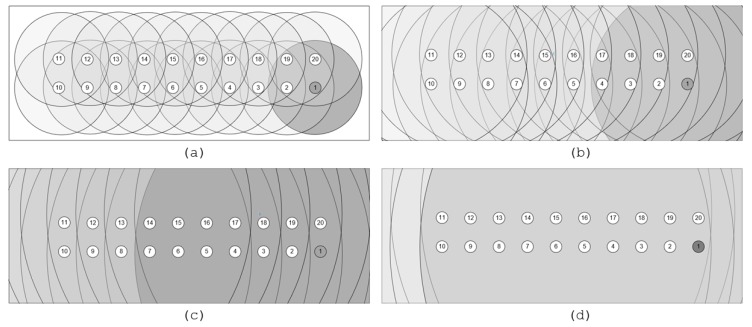
Influence of transmission range in the number of neighbors.

Figure 17 illustrates the influence of the transmission range on the number of neighbors for each node and the consequent variation of the hop number between the 6LBR and the intermediate and the farthest nodes. In Figure 17a, for the 25-m transmission range, each node has three to six neighbors, increasing to seven to 14 neighbors for the 50-m range (Figure 17b), to 13 to 18 for the 100-m range (Figure 17c) and to 19 neighbors for the 150-m range (Figure 17d). In the latter case, all nodes have direct connectivity to the 6LBR.

In order to simulate different transmission ranges, four COOJA scripts were created in “nogui” mode (without the graphical interface). The produced logs contain transmission timestamps, allowing the calculation of the latency. Packets were captured by the COOJA plugin named radiologger-headless, the records of which are compatible with the Wireshark tool. For each range, the simulation is performed thirty times. Table 5 compares the number of SD6WSNP messages with respect to RPL messages, the last being present in both cases. It can be observed that the increase caused by SD6WSNP messages is lower for larger transmission ranges. This is due to the smaller number of entries in the flow tables because of the reduction of hops in corresponding paths. In the worst case, the increase of SD6WSNP messages is 5.71%, which is acceptable considering the benefits that SDN can bring 6LoWPAN. Please note that the overhead incurred due to SD6WSNP messages (including all notifications) is reported in Table 5.

**Figure 18:**
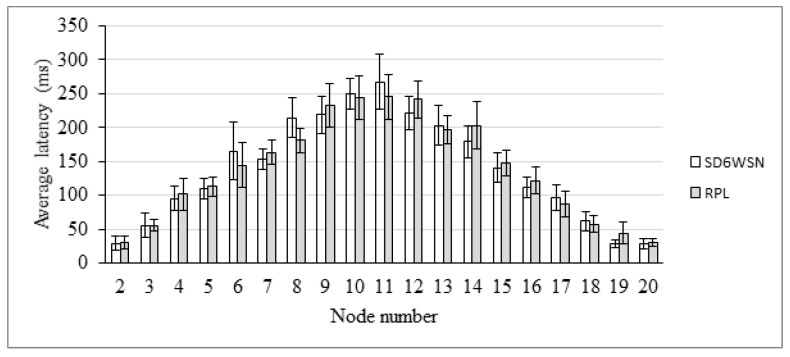
Average latency for the 25 m-range scenario.

**Figure 19:**
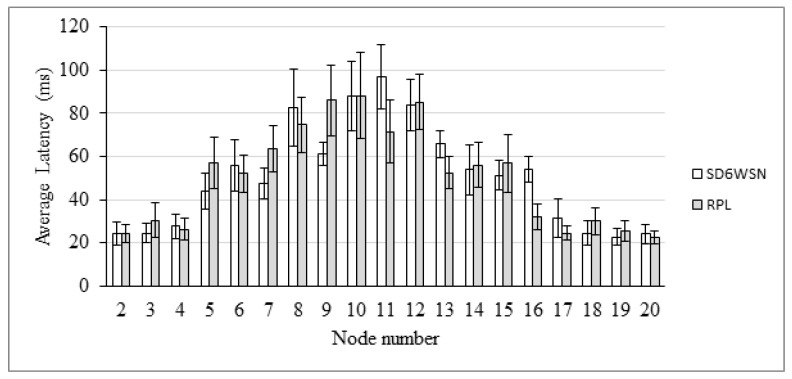
Average latency for the 50 m-range scenario.

**Figure 20:**
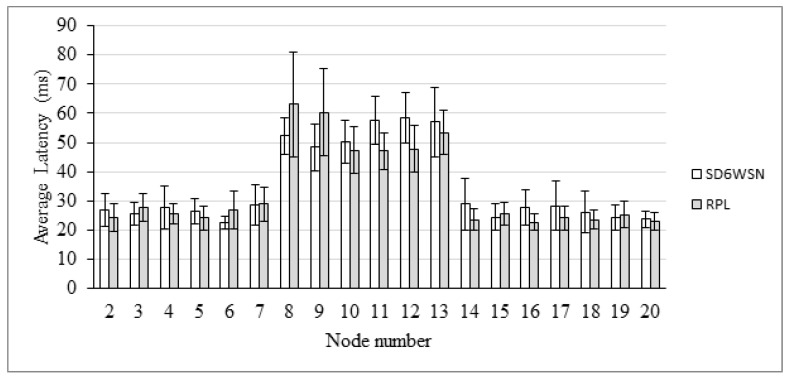
Average latency for the 100 m-range scenario.

**Figure 21:**
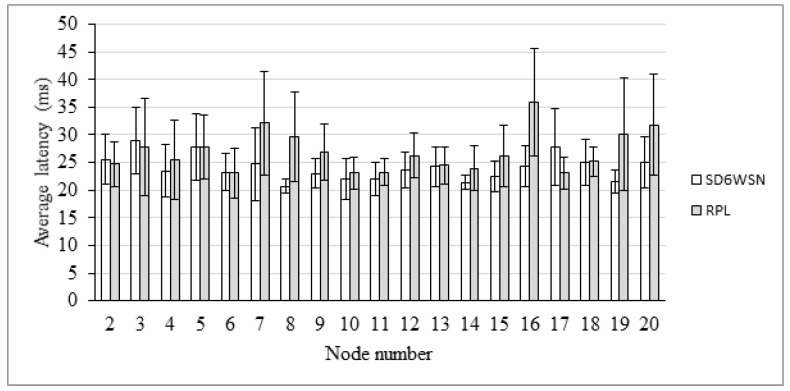
Average latency for the 150 m-range scenario.

End-to-end delay is also evaluated in the AMI scenario. The comparison between RPL and SD6WSN can be observed in Figure 18, Figure 19, Figure 20 and Figure 21. Because both protocols compute similar routes, they perform similarly. Latencies are statistically equivalent in the four transmission ranges.

### 5.3. Peer-to-Peer Scenario

**Figure 22:**
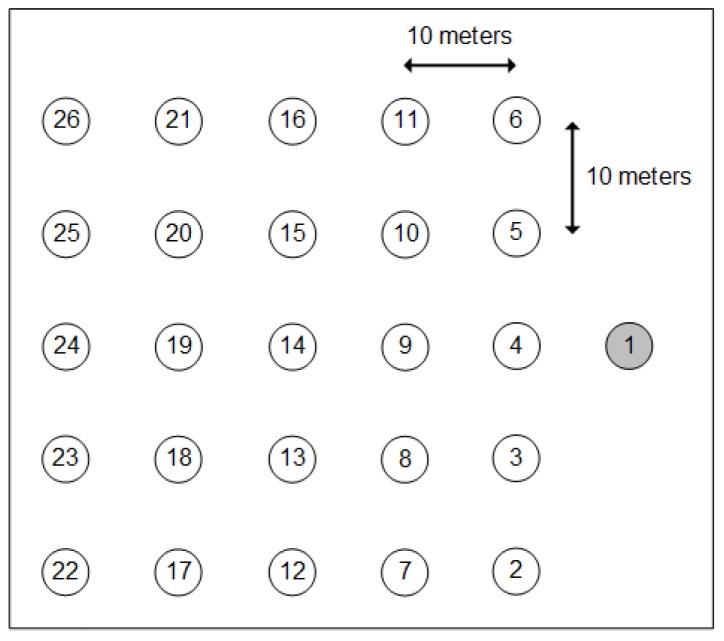
The 5 × 5 m grid topology.

The purpose of this experiment is to evaluate the performance of communication between peer nodes, with the paths between source and destination defined by the SD6WSN peer-to-peer application in comparison to the paths obtained by RPL. The latter optimizes the communication between the nodes and 6LBR, causing peer-to-peer paths to possibly involve a deviation to a common ancestor. The SD6WSN application has the unified view of the topology and uses a centralized shortest path algorithm to compute the paths, installing the corresponding flows along them. The simulation scenario consists of 25 nodes in a grid-type topology, plus the 6LBR, highlighted in gray in Figure 22. All nodes have been compiled with a server and a client, so they can send and receive packets from any node in the grid, simulating the traffic between peer-nodes.

**Figure 23:**
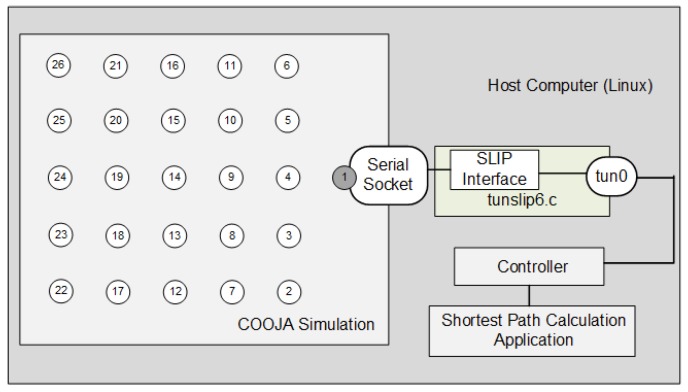
COOJA simulation and SD6WSN controller integration.

The number of possible source-destination pairs is 600 for the topology of Figure 22. Among them, sixty pairs are randomly selected (offline) in three groups of 20. In each group, the origin is not repeated, but the destination is chosen without restrictions, except when the destination is the origin itself. An auxiliary CoAP resource is compiled to each node so that it can receive a configuration message informing its destination. Each source node sends 30 packets with a 20-byte payload, one every 10 s. The simulation scenario is shown in Figure 23. Paths are computed ordinarily by RPL. The SD6WSN application computes the paths according to the unified topology, installing the corresponding flows on the nodes along them. In the case of topology changes, the SD6WSN application recomputes the affected paths and proceeds with the changes of the corresponding flow tables.

**Figure 24:**
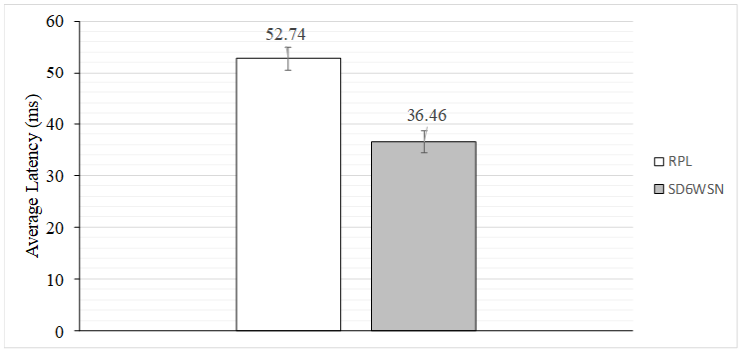
Average latency comparison between RPL and SD6WSN WSNs.

COOJA is executed in “nogui” mode, using the UDGM model, with transmission range set to 25 m and interference range to 50. The quality of wireless media is set to 75% of success in transmission and 100% in reception. We performed 10 simulations, each with three rounds of 20 origin-destination pairs. In each round, each origin node sends 30 UDP packets, with the random seed of the UDGM model changed. Figure 24 shows the average latencies with the 95% confidence interval. It can be observed that average latency is 30.87% lower in SD6WSN when compared to RPL. This result was expected because RPL is optimized for traffic between nodes and the 6LBR; however, the gain is significant and indicates the potential of the proposed architecture.

## 6. Conclusions

This paper presented the SD6WSN architecture, developed to control data plane traffic in 6LoWPAN according to the SDN approach. The design of SD6WSN considers the specific characteristics of WSN devices, such as low data transfer rate, high latency, packet loss and low processing power. It also sought to take advantage of the flexibility provided by flow-based forwarding of the SDN approach, which allows the development of specific networking applications capable of routing packages in the most appropriate way depending on particular requirements.

The Contiki operating system served as the basis for implementing the proposed architecture in motes used in real-world implementations. We provide in this paper the detailed description of the SD6WSN implementation in Contiki motes. We also provide the results of two performance evaluation experiments in the COOJA emulator. The overhead introduced by SD6WSNP messages was evaluated in an AMI network scenario with four distinct propagation settings. The results indicate that the introduced overhead is not excessive, given the advantages that the SDN approach can bring. In the second experiment, a grid-topology was used to compare the average latency of peer-to-peer communication. In this scenario, the average communication latency of SD6WSNP was significantly lower than that obtained with RPL.

Due to the current memory limitation of the motes, some points have not yet been addressed in the SD6WSN implementation. We are working especially on two of them: buffering the incoming messages in situations that generate a packet-in notification and the securitization of SD6WSNP messages. In future works, with motes with greater memory capacity, we will include and evaluate both.

SD6WSN opens up many possibilities for research. Because future SD6WSN networking applications will have a comprehensive and reasonably accurate real-time view of the state of the wireless network, they could plan the flows taking into account this unified view. Moreover, because SD6WSN applications can act on the motes, for example by changing their transmission power or channel, it is possible to configure flows that would meet specific latency or packet loss requirements. Furthermore, because the routing behavior is fully determined by the controller, it is possible to configure flows to isolate the traffic among specific nodes, making it easier to improve security requirements.

## Figures and Tables

**Table 1:** Match field of SD6WSN flow table entry.

Match	Length (Bits)	Description
ipv6src	128	Origin address (IPv6)
srcmask	8	Mask of origin address (default/128))
ipv6dst	128	Destination address (IPv6)
dstmask	8	Mask of destination address (default/128)
srcport	16	Origin transport port
dstport	16	Destination transport port
ipproto	8	IP protocol (19-UDP, 6-TCP, ICMPv6)

**Table 2:** Action field of the SD6WSN flow table entry.

Action	Length (Bits)	Description
type	8	0, Forward the packet to the next-hop through the data plane
		1, Drop the packet
		2, Forward the packet to the controller through the control plane
nhipaddr	128	Next-hop IPv6 address (required parameter for Type 0)
rfpwr	8	Transmission power (optional parameter for Type 0)

**Table 3:** SD6WSNP messages.

Message	Method	Observe	Process
node-mod	GET	Required	Topology discovery and management
info-get	GET	Required in 1st message, optional in others	Topology discovery and management
flow-mod	PUT	Required	Flow control
packet-in	GET	Required	Flow control

**Table 4:** Resource-constrained device classes [32].

Name	Data Area Size (RAM)	Code Area Size (Flash)
Class 0, C0	<<10 kbytes	<<100 kbytes
Class 1 C1	∼10 kbytes	∼100 kbytes
Class 2, C2	∼50 kbytes	∼250 kbytes

**Table 5:** Increasing of control messages due to SD6WSN.

Range	RPL Messages	SD6WSN Messages	Increasing of Control Messages (%)
25 m	30,412	1736	5.71
50 m	13,245	239	1.80
100 m	9186	160	1.74
150 m	6771	73	1.08
